# Identifying the chiral *d*-wave superconductivity by Josephson *φ*_0_-states

**DOI:** 10.1038/srep43899

**Published:** 2017-03-07

**Authors:** Jun-Feng Liu, Yong Xu, Jun Wang

**Affiliations:** 1 Department of Physics, South University of Science and Technology of China, Shenzhen 518055, China; 2 Department of Physics, Southeast University, Nanjing 210096, China

## Abstract

We propose the Josephson junctions linked by a normal metal between a *d* + *id* superconductor and another *d* + *id* superconductor, a *d*-wave superconductor, or a *s*-wave superconductor for identifying the chiral *d* + *id* superconductivity. The time-reversal breaking in the chiral d-wave superconducting state is shown to result in a Josephson *φ*_0_-junction state where the current-phase relation is shifted by a phase *φ*_0_ from the sinusoidal relation, other than 0 and *π*. The ground-state phase difference *φ*_0_ and the critical current can be used to definitely confirm and read the information about the *d* + *id* superconductivity. A smooth evolution from conventional 0-*π* transitions to tunable *φ*_0_-states can be observed by changing the relative magnitude of two types of *d*-wave components in the *d* + *id* pairing. On the other hand, the Josephson junction involving the *d* + *id* superconductor is also the simplest model to realize a *φ*_0_- junction, which is useful in superconducting electronics and superconducting quantum computation.

Much interest has been attracted to recent theoretical predictions that the superconducting pairing driven by strong electron correlation in materials with three- and sixfold rotational lattice symmetries favors topological chiral *d* + *id* symmetry. This symmetry is close to the *d*-wave superconducting pair in high temperature superconductors, but time-reversal broken. The materials which have been proposed to be chiral *d*-wave superconductors include graphene and silicene^1^,^2^,^3^, MoS_2_^4^,^5^,^6^, In_3_Cu_2_VO_9_^7^, SrPtAs^8^,^9^, and bilayer SrIrO_3_^10^,^11^. But the experimental identification is still lacking. One of the experimental method to verify the chiral *d*-wave superconductivity is detecting the edge supercurrent induced by the topologically nontrivial superconducting order. But so far, no definite evidence of edge supercurrent is observed. Also, even theoretically, whether chiral *d*-wave supports an edge supercurrent still remains controversial^12^. Therefore, how to identify this novel superconducting order in experiments is of significant importance to further develop the microscopic theory of high temperature superconductors. The Josephson effect is another powerful method to identify the superconducting pair, should be promising to definitely identify the chiral *d*-wave superconductivity.

The efforts to detect the chiral *d*-wave order by Josephson effect are currently focusing on the signal of the critical current of the Josephson junction which varies with the superconducting order or other junction parameters. But the critical current, the amplitude of supercurrent, includes only indirect information on the pair symmetry, and can not definitely identify the chiral *d*-wave order. By contrast, the ground-state phase difference of the Josephson current, namely, the so-called Josephson *φ*_0_-states contains more ample and direct information on the pair potential, thus should be more promising to definitely identify the chiral *d*-wave pairing. Therefore, the theoretical and experimental investigation in the relation between the ground-state phase difference and the chiral *d*-wave pairing become important. To our best knowledge, the effort in this way is still blank Fig. 1.

In the Josephson *φ*_0_-state, or the anomalous Josephson effect, the current-phase relation (CPR) has a phase shift *φ*_0_ compared with the conventional CPR, namely, *I*(*φ*) = *I*_*c*_sin(*φ* − *φ*_0_)^13^,^14^,^15^,^16^,^17^,^18^,^19^,^20^. The ground-state phase difference *φ*_0_ is neither 0 nor *π* in general, and tunable by the junction parameters. Such a *φ*_0_-state has been predicted in Josephson junctions with coexisting exchange field and spin-orbit coupling^14^,^15^,^16^,^17^,^19^, multilayer ferromagnets^18^,^20^, noncentrosymmetric superconductors^21^,^22^,^23^, and topological edge or interface states^24^,^25^,^26^. Very recently, the first experimental demonstration of a *φ*_0_-junction has been reported in a quantum dot junction by use of a quantum interferometer device^27^. These *φ*_0_-junctions with tunable ground-state phase difference may have applications in superconducting computer memory components^28^, superconducting phase batteries and rectifiers^29^, as well as flux- or phase-based quantum bits^30^. Among various ways to achieve a *φ*_0_-junction, the junctions involving chiral *d*-wave superconductors should be the simplest one because the time-reversal symmetry is already broken in chiral *d*-wave pairing.

In this study, we investigate the Josephson junctions linked by a normal metal between a *d* + *id* superconductor and another *d* + *id* superconductor, a *d*-wave superconductor, or a *s*-wave superconductor (see Fig. 1). Anomalous Josephson effect appears as a result of the broken time-reversal symmetry in the *d* + *id* superconductor. The ground-state phase difference *φ*_0_ other than 0 and *π* should be the definite evidence of the *d* + *id* pairing. Furthermore, the ground-state phase difference and the critical current can be used to determine the ratio between two types of *d*-wave components in the *d* + *id* superconductor. The demonstration of a smooth evolution from conventional 0-*π* transitions to tunable *φ*_0_-states is also interesting.

The paper is organized as follows. In Sec. II we present the model Hamiltonian and introduce the method to solve the CPR. The numerical results and relevant discussion of three types of junctions will be given in Sec. III. Finally, the conclusion will be given in Sec. IV.

## Model and Methods

We begin with the BdG Hamiltonian of a two-dimensional *d* + *id* superconductor with parabolic spectrum in the normal state. The particular form of the spectrum may not take much effect in the ground-state phase difference of the Josephson junction, but the phase of the pair potential does. The spin degree of freedom is also not important and ignored here. Then the simplest BdG Hamiltonian of a *d* + *id* superconductor can be written as


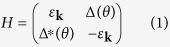


where 
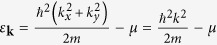
 is the kinetic energy measured from the chemical potential *μ*. The *d* + *id* pair potential is


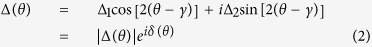


with


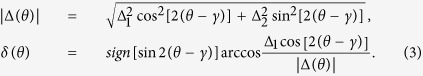


Here *θ* is the injection angle satisfying tan*θ* = *k*_*y*_/*k*_*x*_, *γ* is is the angle between the *x*-direction and the *α* axis of the superconductor. Δ_1_ and Δ_2_ are two positive real numbers, denoting the amplitudes of two kinds of d-waves. It is clearly shown that an additional phase *δ*(*θ*) emerges in the pair potential and depends on the injection anlge.

For a Josephson junction between two d + id superconductors, the pair potential can be approximately described by two step functions Δ(*x*) = [Δ_*L*_Θ(−*x*)*e*^*iφ*/2^ + Δ_*R*_Θ(*x* − *L*)*e*^−*iφ*/2^] where *L* is the length of the normal layer and *φ* is the macroscopic phase difference between two superconductors. Similar to Eq. (delta), the left (right) pair potential Δ_*L*_ (Δ_*R*_) reads 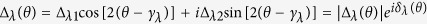 with *λ* = *L* or *R* denoting the left or right superconductor respectively. For simplicity, we assume that the momentum component in the *y*-direction is conserved. Then the eigen wavefunctions in two superconductors can be written as


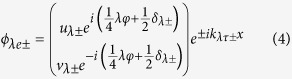


for electron-like quasiparticles and


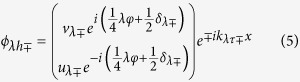


for hole-like quasiparticles where 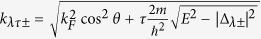, and 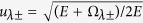, 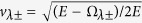 with 
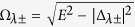
. Here 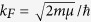 is the Fermi wave vector, *λ* = 1 (−1) for the left (right) superconductor, *τ* = 1 (−1) for electron-like (hole-like) quasiparticles. Δ_*λ*±_ = Δ_*λ*_(±*θ*) with Δ_*λ*+_ (Δ_*λ*−_) being the pair potential for right-going electron-like and left-going hole-like (left-going electron-like and right-going hole-like) quasiparticles. *δ*_*λ*±_ = *δ*(±*θ, γ*_*λ*_) according to Eq. (3).

The eigen wavefunctions can be easily solved for the normal layer. Then the scattering problem can be solved by considering the boundary conditions at two interfaces. Each interface gives a scattering matrix, from which the reflection matrix of the right-going (left-going) incident particles *R*_1_ (*R*_2_) can be abstracted in the normal layer. To calculate the Josephson current, we can work out the Green’s function *G*(*z, z*’, *E*) in the normal layer which is made of the reflection matrices *R*_1_ and *R*_2_^31^. Then the Josephson current in the normal layer can be evaluated by


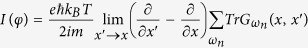


where 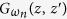 is the Matsubara-Green’s function with the Matsubara frequencies *ω*_*n*_ = *πk*_*B*_*T*(2*n* + 1), *n* = 0, ±1, ±2, 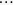. By integrating over the injection angle *γ*, the total Josephson current in two dimension is


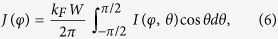


where *W* is the transversal width of the junction.

## Results and Discussion

Next, we present the numerical results and relevant discussion of three types of junctions involving chiral *d*-wave superconductors. We focus on the ground-state phase difference and critical current of the CPR. The interface transparency is found to neither change the ground-state phase difference, nor change the relative magnitude of critical current, but the absolute value of critical current. Therefore, the results about the effect of interface barriers is not presented here.

### Junction between two *d* + *id* superconductors

**Figure 2:**
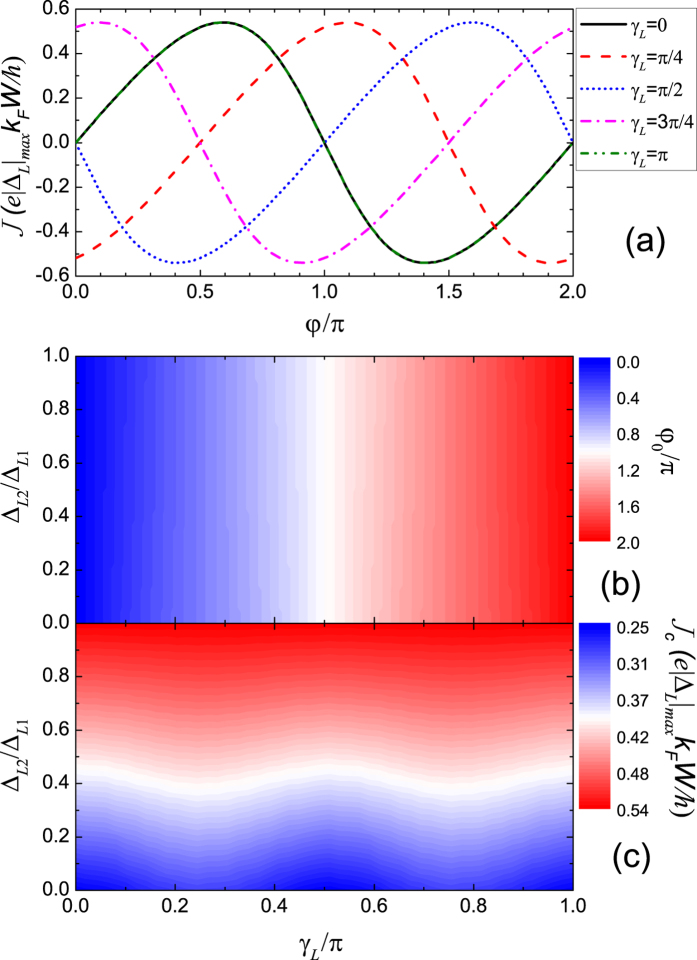
Josephson current for the Junction between two *d* + *id* superconductors with different directions of the *α*-axis. (**a**) CPR for various *γ*_*L*_ with fixed Δ_*L*2_/Δ_*L*1_ = 1. (**b**) The ground-state phase difference and (**c**) the critical current as functions of Δ_*L*2_/Δ_*L*1_ and *γ*_*L*_ are shown in the contour plots. The temperature *T* = 0.5*T*_*c*_ with *T*_*c*_ the critical temperature. Δ_*L*1_ = Δ_*R*1_ = Δ_*R*2_ = 10^−3^ *μ, γ*_*R*_ = 0. |Δ_*L*_|_*max*_ = max(Δ_*L*1_, Δ_*L*2_).

For the Junction between two *d* + *id* superconductors with different directions of the *α*-axis, the Josephson current is shown in Fig. 2. In the simple case Δ_*L*1_ = Δ_*L*2_ = Δ_*R*1_ = Δ_*R*2_, the additional phase in the pairing potential for the left and right superconductor *δ*_*λ*_(*θ*) = 2(*θ* − *γ*_*λ*_) according to Eq. (3). Then the additional phase difference is 2(*γ*_*R*_ − *γ*_*L*_), which means the ground-state phase difference is 2*γ*_*L*_ when *γ*_*R*_ = 0. It is exactly the case shown in Fig. 2(a) where the nearly sinusoidal CPR just moves to the right with increasing *γ*_*L*_. The ground-state phase difference increases smoothly, while the shape and amplitude of the CPR keep unchanged. In the other limit Δ_*L*2_ = 0, Δ_*L*_(*θ*) = Δ_*L*1_cos[2(*θ* − *γ*_*L*_)]. The left superconductor becomes a *d*-wave superconductor and the additional phase is 0. However, |Δ_*L*_(*θ*)| reaches its maximum at *θ* = *γ*_*L*_, 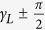, or *γ*_*L*_ − *π*. It implies that the Josephson current is dominated by the components related to these two incident angles. For these two components, the pair potential changes sign for both left and right superconductors. Then the ground-state phase difference is nearly still 2*γ*_*L*_ because the additional phase for the right superconductor is 2*θ* with *γ*_*R*_ = 0. As shown in Fig. 2(b), the ground-state phase difference *φ*_0_ nearly keeps unchanged with varied ratio Δ_*L*2_/Δ_*L*1_ and just equals 2*γ*_*L*_ with increasing *γ*_*L*_. The critical current *J*_*c*_ is shown in Fig. 2(c). With decreasing ratio Δ_*L*2_/Δ_*L*1_, the critical current decreases because |Δ_*L*_(*θ*)| decreases for all the incident angles *θ* except for the angles at which |Δ_*L*_(*θ*)| takes the maximum. Note that with a fixed Δ_*L*2_/Δ_*L*1_ smaller than 1, the critical current oscillates with a period of 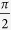 with increasing *γ*_*L*_. This oscillation is due to the factor cos*θ* in Eq. (6) and the period 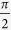 is attributed to the four-fold rotational symmetry of *d* + *id* pairing. With decreasing ratio Δ_*L*2_/Δ_*L*1_ from 1 to 0, the amplitude of the oscillation increases from 0.

### Junction between a *d* + *id* superconductor and a *d*-wave superconductor

**Figure 3:**
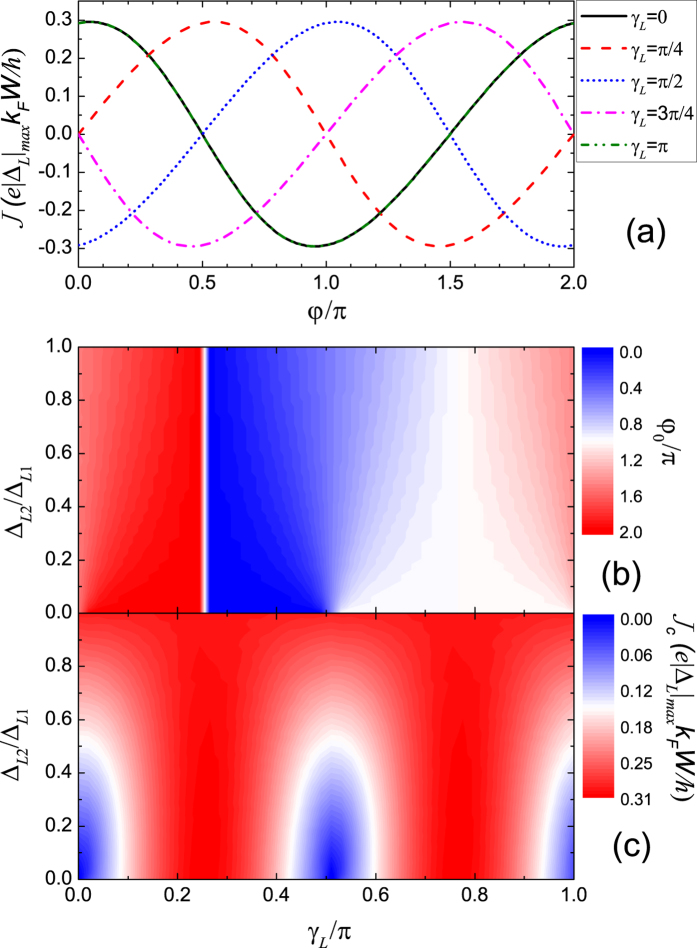
Josephson current for the Junction between a *d* + *id* superconductors and a *d*-wave superconductor. (**a**) CPR for various *γ*_*L*_ with fixed Δ_*L*2_/Δ_*L*1_ = 1. (**b**) The ground-state phase difference and (**c**) the critical current as functions of Δ_*L*2_/Δ_*L*1_ and *γ*_*L*_ are shown in the contour plots. *T* = 0.5*T*_*c*_, Δ_*L*1_ = Δ_*R*1_ = 10^−3^ *μ*, Δ_*R*2_ = 0, *γ*_*R*_ = *π*/4.

Figure 3 shows the Josephson current through the junction between a *d* + *id* superconductor and a *d*-wave superconductor. The *d*-wave pairing in the right superconductor is chosen to be a *d*_*xy*_-wave pairing by setting Δ_*R*2_ = 0 and 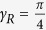. According to the above discussion, the right *d*_*xy*_-wave pairing decides that the dominant components in the Josephson current come from the incident angles 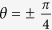. When Δ_*L*2_/Δ_*L*1_ = 1, the additional phases in Δ_*L*_(*θ*) for the two components are 
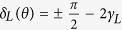
, which leads to the ground-state phase difference 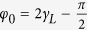 by taking into account of the sign change in *d*_*xy*_-wave pairing. The numerical results on *φ*_0_ shown in Fig. 3(a,b) verify this discussion. Because |Δ_*L*_(*θ*)| is independent of *θ* and *γ*_*L*_ for Δ_*L*2_/Δ_*L*1_ = 1, the critical current is also independent of *γ*_*L*_ as shown in Fig. 3(a,c). In the other limit Δ_*L*2_ = 0, the left superconductor reduces to a *d*-wave superconductor and its additional phase becomes 0. Then the anomalous Josephson effect disappears and *φ*_0_ must be either 0 or *π*. With increasing *γ*_*L*_, the junction experiences conventional 0-*π* transitions which is accompanied with heavy oscillations in the critical current. The oscillations in the critical current come from the varying angles between the *α*-axes of two *d*-wave superconductors. Specifically, the junction is a 0-junction for 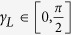 and turns into a *π*-junction for 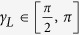, accompanied with minima in *J*_*c*_ at the transition points *γ*_*L*_ = 0, 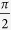, *π*. It is interesting to note the smooth transition between these two limiting cases Δ_*L*2_/Δ_*L*1_ = 0 and 1. With the changing of Δ_*L*2_/Δ_*L*1_ from 1 to 0, *φ*_0_ increases more and more nonuniformly with increasing *γ*_*L*_, at the same time accompanied with more and more heavy oscillations in *J*_*c*_, and finally evolves into a conventional 0-*π* transition.

### Junction between a *d* + *id* superconductor and a *s*-wave superconductor

The situation is similar for the junction between a *d* + *id* superconductor and a *s*-wave superconductor. The *s*-wave pairing in the right superconductor is described by setting Δ_*R*_(*θ*) = Δ_*R*_ where the additional phase *δ*_*R*_(*θ*) = 0. When Δ_*L*2_/Δ_*L*1_ = 1, the additional phase in Δ_*L*_(*θ*) is *δ*_*L*_(*θ*) = 2(*θ* − *γ*_*L*_). The angle-resolved Josephson current in the first harmonic approximation is *I*(*θ*) ∝ |Δ_*L*_|Δ_*R*_sin(*φ* + 2*θ* − 2*γ*_*L*_). The total Josephson current is


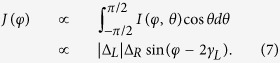


**Figure 4:**
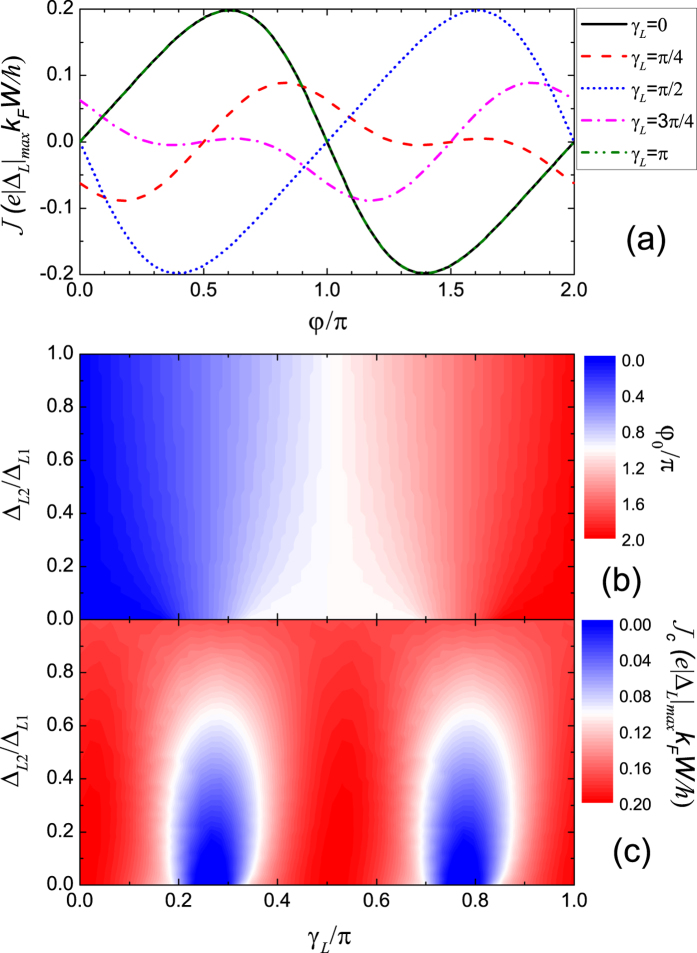
Josephson current for the Junction between a *d* + *id* superconductors and a *s*-wave superconductor. (**a**) CPR for various *γ*_*L*_ with fixed Δ_*L*2_/Δ_*L*1_ = 0.3. (**b**) The ground-state phase difference and (**c**) the critical current as functions of Δ_*L*2_/Δ_*L*1_ and *γ*_*L*_ are shown in the contour plots. *T* = 0.5*T*_*c*_, Δ_*L*1_ = Δ_*R*_ = 10^−3^ *μ*.

This conclusion of *φ*_0_ = 2*γ*_*L*_ is consistent with the numerical result shown in Fig. 4(b). When Δ_*L*2_/Δ_*L*1_ = 0, the junction reduces to a *d*-wave/normal metal/*s*-wave junction. Only the conventional 0-*π* transition is possible due to the sign change in the *d*-wave pairing with increasing *γ*_*L*_. The evolution from the tunable *φ*_0_-state for Δ_*L*2_/Δ_*L*1_ = 1 to conventional 0-*π* transition for Δ_*L*2_/Δ_*L*1_ = 0 is similar to that discussed in Fig. 3. For the general case between Δ_*L*2_/Δ_*L*1_ = 1 and 0, the change of *φ*_0_ with increasing *γ*_*L*_ is simultaneously accompanied with the oscillation in *J*_*c*_. The oscillation in *J*_*c*_ is achieved by second and higher harmonic terms as shown in Fig. 4(a).

Finally, we comment on the experimental feasibility of observing the *d* + *id* superconductivity. In view of the fact that there are various possible paring mechanisms^32^,^33^ in graphene and graphene-like materials, we suggest that SrPtAs should be the most promising candidate to observe the *d* + *id* pairing. The experimental challenge lies in the observing the ground-state phase difference, and can be met by employing the superconducting quantum interference device^27^. We argue that the slight nonmagnetic impurities do not affect the ground-state phase difference qualitatively if only the *d* + *id* pairing is not destroyed. But the interplay between the magnetic impurities and the superconductivity is complicated and beyond the topic of this paper, and may be the next aim in our further study. Otherwise, we employ a quadratic dispersion relation to describe the quasiparticles in the normal state for the *d* + *id* paired superconducting leads. This quadratic dispersion is obviously invalid for graphene and graphene-like materials. But the result for the ground-state phase difference will not be changed because it is only affected by the phase of the pair potential. The critical current will be qualitatively unchanged but quantitatively modified if the linear dispersion is adopted.

## Conclusion

In conclusion, we study the anomalous Josephson effect in junctions with chiral *d* + *id* superconductor induced by the time-reversal breaking of superconducting order. The ground-state phase difference *φ*_0_ other than 0 and *π* is predicted and should be the definite evidence of the *d* + *id* pairing. The ground-state phase difference and the critical current are shown to depend on the ratio between two types of *d*-wave components and the direction of the *α*-axis of the *d* + *id* superconductor. The demonstration of a smooth evolution from conventional 0-*π* transitions to tunable *φ*_0_-states advances the understanding of Josephson effect. And the simple *φ*_0_-junction consisting of the *d* + *id* superconductor is important to applications in superconducting electronics and superconducting quantum computation.

## Additional Information

**How to cite this article:** Liu, J.-F. *et al*. Identifying the chiral *d*-wave superconductivity by Josephson φ_0_-states. *Sci. Rep.* **7**, 43899; doi: 10.1038/srep43899 (2017).

**Publisher's note:** Springer Nature remains neutral with regard to jurisdictional claims in published maps and institutional affiliations.

## Figures and Tables

**Figure 1:**
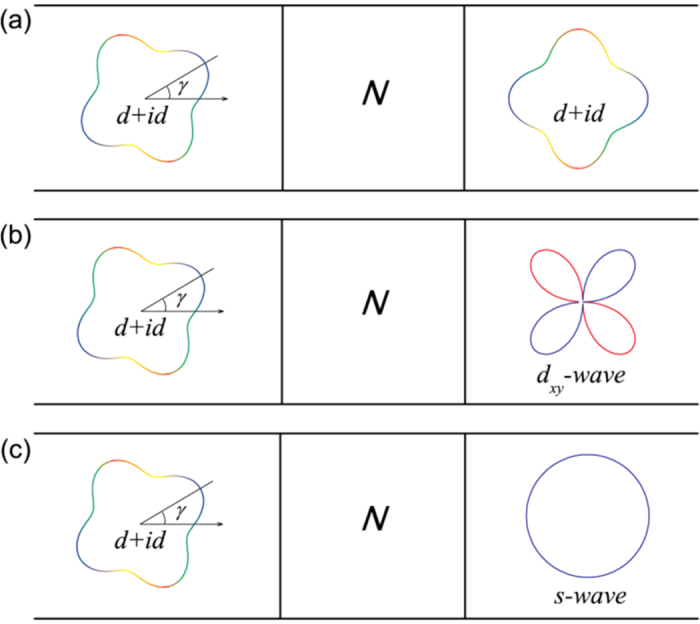
Schematic diagrams of Josephson junctions linked by a normal metal between a *d* + *id* superconductor and (**a**) another *d* + *id* superconductor, (**b**) a *d*-wave superconductor, and (**c**) a *s*-wave superconductor. The junctions lie in the *x*-*y* plane and the transport is along the *x*-direction.
